# Piloting an International Comparison of Readily Accessible Online English Language Advice Surrounding Responsible Cat Ownership

**DOI:** 10.3390/ani13152434

**Published:** 2023-07-27

**Authors:** Reece J. Dalais, Michael C. Calver, Mark J. Farnworth

**Affiliations:** 1 School of Veterinary Medicine, Murdoch University, 90 South Street, Murdoch, WA 6150, Australia; 2 Environmental and Conservation Sciences, Murdoch University, 90 South Street, Murdoch, WA 6150, Australia; 3 Department of Animal Health, Behaviour and Welfare, Harper Adams University, Newport TF10 8NB, UK; 4 The Jeanne Marchig International Centre for Animal Welfare Education, Royal (Dick) School of Veterinary Studies, Easter Bush, Midlothian EH25 9RG, UK

**Keywords:** responsible cat ownership, cat husbandry, cat containment, domestic cat management, registration, microchipping, sterilisation, neutering, desexing, environmental and behavioural enrichment, *Felis catus*

## Abstract

**Simple Summary:**

Cats coexist in almost all places of human habitation. An English-language internet search identified 16 different recommendations for responsible ownership across 10 nations from 58 different webpages. Most webpages were attributed to Australian local governments, so recommendations may lack local nuance in other countries. More than half the webpages recommended registration and microchipping (65.5%), desexing (65.5%), and containment (60.3%). Both Australia and New Zealand showed majority support for containment, possibly because there is significantly higher agreement in Australia and New Zealand that cats threaten valued wildlife in cities, towns, and rural areas. There were, however, few clear recommendations from these countries for environmental and behavioural enrichment for pet cats in households. Unlike the Oceanian nations, in the webpages explored, all other countries recommended improved understanding and provision for cat needs, but with few recommendations for containment. Different welfare considerations are informing attitudes towards cat ownership internationally. Encouraging containment, a responsible cat ownership practice with benefits for cats and wildlife, may be more likely to succeed outside Oceania if cat welfare is emphasised instead of wildlife protection. Within Oceania, more attention could be given to enhancing the well-being of contained cats.

**Abstract:**

Cats are popular companion animals globally. While the general academic definition of responsible cat ownership is agreed upon, committing to responsible cat ownership is multifaceted, often reflecting regional priorities and values. Utilising a virtual private network (VPN), an English-language online search for ‘responsible cat ownership’ was applied from major cities in 10 different nations, accounting for five different geographic regions and nine different geographic sub-regions. Data were extracted from the first 20 webpages of each search and included author affiliation, country of webpage origin, and all recommendations towards responsible cat ownership. Searches identified 58 different webpages, 142 duplicate results, and 16 different recommendations. Both before (60.5%) and after (58.6%) duplicate exclusion, irrespective of region, most webpages originated from Australia, so recommendations may lack local nuance in other countries. Similarly, local government webpages were the most common author affiliation both before (35.5%) and after (37.9%) duplicate exclusion—moreover, most Australian webpages were authored by local government (55.9%). More than half of all webpages recommended registration and microchipping (65.5%), desexing (65.5%), and containment (60.3%), probably due to the predominance of local government and Australian webpages online—reflecting Australia’s strong legislative stance. Both Australia and New Zealand showed majority recommendations for containment but not for environmental and behavioural enrichment in households. This may be partially explained by the significantly higher agreement in Australia and New Zealand that cats threaten valued wildlife in cities, towns, and rural areas. Unlike the Oceanian nations, other countries clearly recommended improved understanding and provision for cat needs, but with little evidence of support for containment. Thus, divergent welfare considerations inform major webpages associated with attitudes towards cat ownership internationally. Encouraging containment, a responsible cat ownership practice with benefits for cats and wildlife, may be more likely to succeed outside Oceania if cat welfare is emphasised instead of wildlife protection. Within Oceania, more attention could be given to enhancing the well-being of contained cats.

## 1. Introduction

There have been pet cats (*Felis catus*) on all continents—including as pets in some Antarctic bases [1,2]. Their popularity as companion animals is second only to dogs in many countries [3,4]. In New Zealand (NZ) [5], Japan [6], and Russia [7], cats are more popular than dogs. Where they occur, cats typically have higher population densities (e.g., >100/km^2^) than dogs [3,4,8]. Cat husbandry practices vary, leading to a plethora of terms to describe their movements in relation to human populations. Here, we follow [9] in broadly defining cats as pets (which may be contained, sometimes contained but with occasional freedom to range, or completely free-ranging with no constraints on their movements), stray (associated with human habitation and receiving intentional or unintentional human subsidies), and feral (self-sustaining without any human subsidy). We do acknowledge, though, that these terms have different emotive connotations for different cohorts of people [9,10,11,12].

We take responsible cat ownership to be a commitment to perform various duties, sometimes specified by legislation, to satisfy a cat’s behavioural, physical, and environmental needs while reducing risks that a cat may pose to the community, other animals, or the environment [13,14,15]. Nevertheless, a commitment to responsible cat ownership is multifaceted, often reflecting the priorities and values of a region and its stakeholders [2,16,17]. Moreover, management decisions and efforts advocated by public authorities do not always translate into ownership practices. Responsible ownership requires owners to (a) identify issues with cat behaviour and/or welfare, (b) accept responsibility for managing cat behaviour and/or welfare, and (c) have knowledge, capacity, and incentives to manage such issues [9]. The theory of planned behaviour (TPB) proposes that attitudes, anteceded by beliefs surrounding the consequences, expectations of important others, and the feasibility of an effort, influence an individual’s intentions to perform a behaviour [18]. Formative to these beliefs and attitudes are personal (emotions and values), informational (experience and knowledge), and social background factors (culture, religion, and ethnicity) [19]. There is support for the utility of the TPB in predicting the influence of attitudes on behaviours towards companion animals [17,20,21], highlighting the probable impact of cultural and religious factors in determining attitudes, commitments, and conflicts within pet ownership. As an example, recent research using behavioural prioritisation in NZ [22] identified that cat containment at night is widely considered acceptable by owners and veterinarians, but that the likelihood of adopting 24 h containment is very low. In this case, the advantages to wildlife are overridden by the perceived cost to the cats’ welfare [22], despite the evidence of increased longevity for cats contained on their owner’s property (e.g., [23,24]). Given that the TPB requires information, we sought to describe what information regarding responsible cat ownership is available online to owners.

Studies of online advice are increasingly common, covering issues as diverse as legal matters [25], health conditions [26], or job-seeking [27], as well as information on animal welfare such as searches for specific diseases of pets [28]. Research methods include broad internet searches [26], studies of exchanges on social media pages or in online forums [29], and experimental designs [27,30]. There is evidence of cultural differences in how people search for or ask questions on social media [29], as well as varying willingness to act on advice based on the nature of that advice or its source [31]. Therefore, it is important to document the source of the advice, its nature, and any evidence of cultural or regional influence.

The aims of this research were to summarise, as a pilot study, the national priorities and recommendations surrounding responsible cat ownership, as indicated by popular webpages available online in English. We focused on owner responsibility rather than cat care because, by our definition, responsibility embraces care of the cat as well as the social dimension of pets in the community (community expectations, relationships with neighbours, legal codes, environmental awareness). We also wished to exclude any considerations of “semi-ownership”, in which people may feed an unowned, free-ranging cat without taking responsibility for other aspects of its care or for its behaviour. The aims were to:

1. Categorise search results by country of search, authorship, and country of webpage origin;
2. Determine the recommendations accessible via Google search and identify if they vary by country of search, authorship, or country of webpage origin;
3. Determine if webpages retrieved using the search term “responsible cat ownership” embraced the social, legal, and environmental aspects of ownership as well as care of the cat;
4. Describe and evaluate a search protocol that could be expanded to consider languages other than English and other search terms relevant to cat management.

## 2. Materials and Methods

Between 1 and 4 January 2022, author Dalais conducted internet searches in English via the Google search engine for “responsible cat ownership.” No other details were used in the search. The searches used a virtual private network (VPN). VPNs enable private browsing by concealing the searcher’s IP address, preventing customised searches based on IP profiles by the search engine. They also hide the user’s virtual location, giving a new one based on the server connecting the user via the VPN, which allows targeted browsing from specific locations. The search was applied to major cities in 10 different nations. While they were selected subjectively, we accounted for five different geographic regions and nine different geographic sub-regions, as defined by the United Nations Statistics Division (https://unstats.un.org/unsd/methodology/m49/, accessed on 20 July 2023). The nations were chosen to give broad global coverage, while cities within them were selected to have a population size of 0.2 million or greater. The same internet browser (Microsoft Edge) and search engine (Google) were used for all searches. All browsing history, download history, cookies, cached images and files, and other site data were cleared between searches, preventing personalised web browsing based on HTTP cookies. The first 20 search results for each search, including personal blogs, webpage PDFs, and academic papers, were included for data extraction and comparison. The first 20 returns were used based on indications that less than 0.4% of search traffic proceeds beyond the 15th search return [32]. Our selections therefore present a realistic landscape of the information retrieved by a typical English-speaking user but do not consider advice available in other languages.

All webpages were classified based on geographical region, sub-region, country of search, and country of origin (i.e., where the author of the webpage was based). English is the national language in six of the 10 countries. The other four were included to indicate the English-language advice occurring there.

Webpages were then scrutinised based on the full text and any relevant hyperlinks. Academic papers were classified by the country of origin of the corresponding author, not the country providing the online platform. Webpage country of origin and literature country of origin are described hereafter as webpage origin. In order to determine authorship for each webpage’s homepage, “about us” or “contact us” were used. Authors were classified into one or more of: (i) federal or state government; (ii) local government; (iii) non-profit organisation; (iv) charity; (v) business; (vi) personal blog; (vii) academic paper; and (viii) any authors with direct veterinary connections. All recommendations towards responsible cat ownership were recorded. Recommendations were extracted and listed. Those recommendations occurring in >50% of any authorship or country category were regarded as carrying majority support, while those occurring in <25% of any authorship or country category were classed as having low support.

Initially, all webpages found in a search from a particular country were used to give an overall picture of the recommendations found in each country and the authors making them. Of course, often the same website is found when searching from multiple countries. Thus, we also considered only different webpages to determine where recommendations originated (i.e., the country of origin of the webpage) and the authors who made those recommendations. We called this approach removal of duplicates and applied it after the initial approach by country. Note that it only removed duplicate webpages rather than identifying a webpage for removal based on the country in which it was found. Thus, results were available in two forms: the webpages found when searching from each country (before duplicate removal) and a separate profile of the unique webpages found in any search (after duplicate removal).

## 3. Results

### 3.1. Authorship and Country of Webpage Origin Found When Searching from Different Countries

The 200 webpages located by the 10 searches identified 58 different pages (including 24 pages that were unique to searches from only one country) and 142 duplicate results. The 58 different webpages originated in six countries (Table 1; see Supplementary Materials S1 for details of the 58 different webpages).

**Figure 1:**
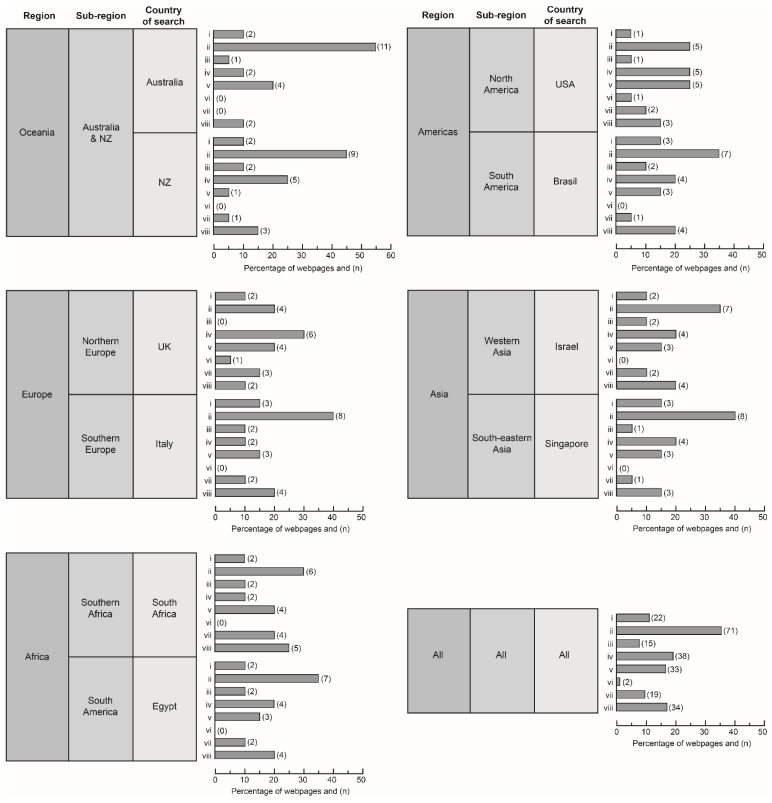
Percentage and number (in parentheses) of webpages from (i) federal or state govt., (ii) local govt., (iii) non-profit organisation, (iv) charity, (v) business, (vi) personal blog, (vii) academic paper, and (viii) authorship with direct veterinary association, grouped by geographic region, sub-region, and country of search. Note that category (viii) may overlap with another category, so totals for a country may exceed 20.

Overall, local government webpages predominated (35.5%) in search results, followed by charities (19.0%) and businesses (16.5%). Only two personal blogs were recorded (1%). Authors with direct veterinary connections comprised 17% of all search results. Local government webpages also predominated for nine different search countries: Australia (55%), NZ (45%), Brazil (35%), Italy (40%), Israel (35%), Singapore (40%) South Africa (30%), Egypt (35%), and the USA (25%). Search results from the USA showed a shared predominance of local government (25%), charities (25%), and businesses (25%). Authorship for the UK search was highest for charities (30%). The only two personal blogs in the dataset occurred in USA and UK searches. Direct veterinary association was highest in South Africa (25%) and lowest in Australia (10%) and the UK (10%). No obvious similarities or differences in authorship were observed within or between geographic regions or sub-regions (Figure 1).

Webpages originated in only six countries. Canada accounted for the webpage origin of 3.5% of all search results, although it was not a search country. Most webpages were from Australia (60.5%), NZ (12%), and the USA (11%). For five of the six countries of origin, the corresponding search yielded the highest number of national webpages (e.g., the number of UK webpages was highest at (7) in the UK search, while the number of USA webpages was highest at (5) in the USA search). This is true for all countries except Singapore, as the most Singaporean webpages were detected in South Africa (2). Australian webpages had the highest proportion, regardless of search country. This proportion was particularly high in Australia (90%), Singapore (70%), and Italy (65%). NZ occupied the second highest proportion in all search countries except Australia (0%), the USA (5%), the UK (0%), and Singapore (5%) (Table 2).

### 3.2. Recommendations for Responsible Ownership Accessible via Google Search from Specific Countries

Sixteen different “responsible cat ownership” recommendations were recorded from all 200 search results. “Registration and microchipping” was cited most frequently (76.5% of 200 webpages) (registration is a synonym for licensing, and is commonly implemented together with microchipping as evidence that it has occurred). Over half of all webpages also recommended desexing (67%) and containment (61.5%). The least commonly encountered recommendations (those accounting for less than 25% of all webpages) were discouraging cruelty to cats (5%), pet insurance (8.5%), the use of cat bibs or bells to discourage hunting (11.5%), discouraging cat abandonment and/or the adoption of stray/rescue cats (16.5%), advocation for ethical breeding practices (18%), and the maximum number of cats per household (20%) (Table 3).

Generally, most recommendations were presented in similar proportions regardless of the country of search (Table 3). If the data are expressed as a percentage of the 20 webpages accessed for each country, advocacy for containment was highest in the Australian search (85%) and lowest in the UK (45%) and USA (50%) searches. The UK search also had the lowest proportion of webpages recommending desexing (35%), which was highest for Australia and NZ (80%); registration and microchipping (50%), which was highest for Australia (90%); and improved disease awareness and prevention for cats (35%), which was highest for the USA (65%). Recommendations towards suitable nutrition for cats were also highest in the USA search (50%) and lowest in Australia (25%), and improved understanding and provision for cat needs were highest in the Israeli and Egyptian searches (60%) and lowest in the Australian search (25%). The proportion of webpages recommending the use of cat bibs or bells was noticeably higher in the NZ search (30%) and lowest in the USA search (5%) (Table 3).

### 3.3. Search Results after Eliminating Duplicate Webpages

The previous sections revealed the patterns of authorship and recommendations that would be found when searching for a specific country. An alternative is to consider only the 58 different webpages, which gives a global perspective on the countries contributing webpages, authorships, and the recommendations made.

Of the six countries of origin of webpages, after duplicate exclusion, most were Australian (34; 58.6%), followed by New Zealand (8; 14%), UK (7; 124%), USA (5; 9%), Singapore (2; 3%), and Canada (2; 3%) (i.e., after duplicate exclusion, no webpage was counted twice). Moreover, most Australian webpages were authored by local governments (56%). Webpages from the USA were predominantly authored by businesses (80%), with a relatively high percentage of veterinary webpages (40%). Half of the webpages from NZ were authored by charities (50%), with local government also prominent (25%). Relatively high percentages of UK webpages were authored by charities (43%) and businesses (29%). An academic paper and a charity contributed to the two Singapore webpages, while a charity and a local government webpage contributed to the two Canadian webpages (Table 4).

Similarly, after excluding duplicate sites, more than half of all webpages recommended registration and microchipping (65.5%), desexing (65.5%), and containment (60.3%). The least common recommendations were discouraging cat abandonment and/or the adoption of stray/rescue cats (19.0%), the use of cat bibs or bells (17.2%), advocation for ethical breeding practices (17.2%), discouraging cruelty to cats (10.4%), and pet insurance (5.2%) (Table 5).

**Figure 2:**
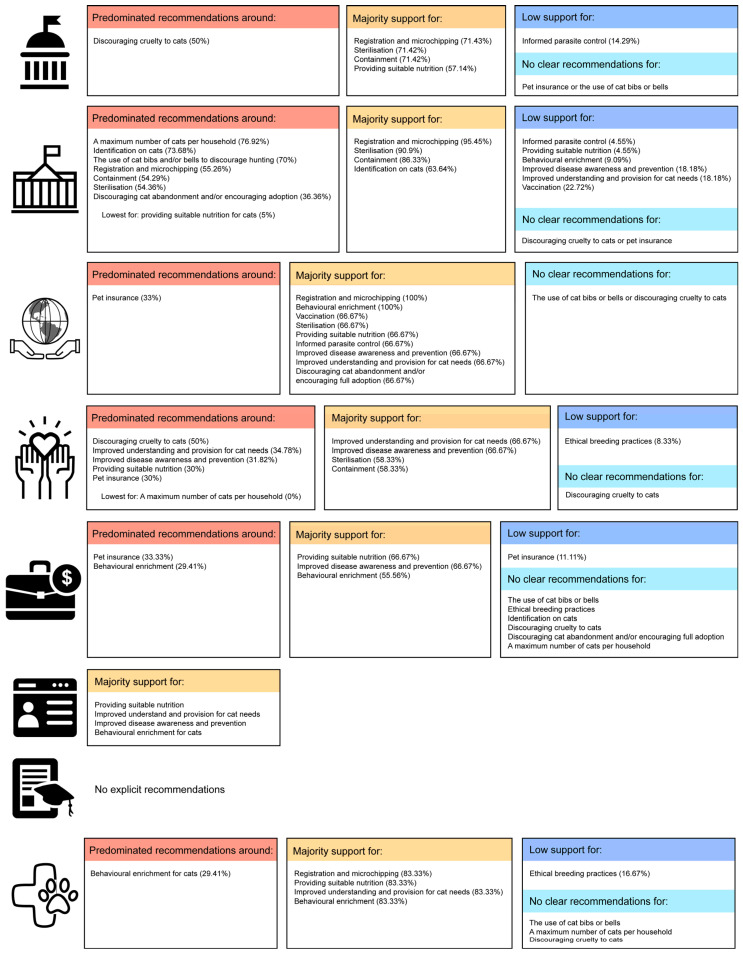
Recommendations by author affiliation, after duplicate exclusion, from the top down: i. Federal or state government (the national government of a country); ii. local government (a local municipality); iii. non-profit organisation (an organisation specifically run not to make a profit. All funds are returned to the work of the organisation); iv. charity (an organisation raising money for animal welfare); v. business (making a profit for private gain); vi. personal blog (regularly updated webpage with one person’s view); vii. academic paper (peer-reviewed journal publication); viii. a direct veterinary association (a webpage of a veterinary association or business). Majority support indicates that >50% of the webpages endorsed the measure; low support indicates that <25% of webpages support). Predominant recommendations form the largest categories, but not necessarily the majority.

**Figure 3:**
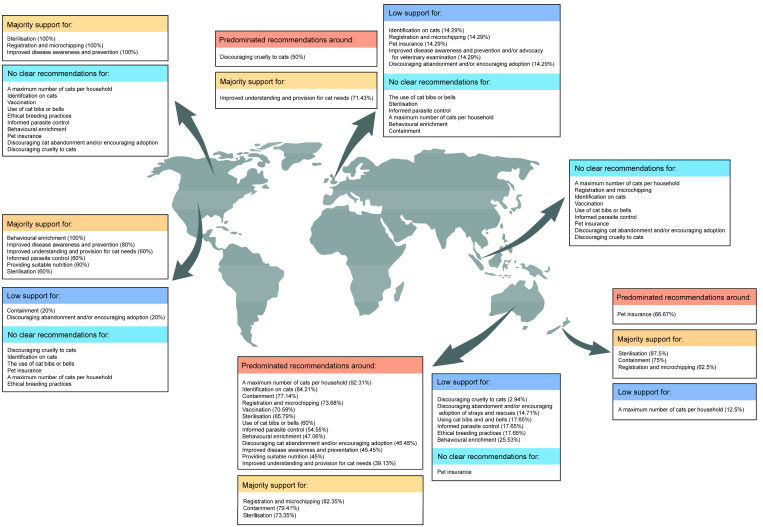
Recommendations by country of webpage origin. Majority support indicates that >50% of the webpages endorsed the measure; low support indicated that <25% of webpages support). Predominant recommendations form the largest categories, but not necessarily the majority.

Recommendations could also be examined by authorship groups (Figure 2) and by the country of origin of webpages (Table 6, Figure 3).

i. Federal and state governments (5 Australia and 1 New Zealand webpage)

Most federal and state government webpages supported registration and microchipping (71.4%), Desexing (71.4%), containment (71.4%), and providing suitable nutrition (57.1%). There was no support for the use of cat bibs or bells or pet insurance, and limited support for informed parasite control (14.3%).

ii. Local government (19 Australia and 2 New Zealand webpages)

Almost all local government sites favour registration and microchipping (95.45%), desexing (90.9%), containment (86.3%), and identification of cats (63.6%). However, less than half recommended a maximum number of cats per household (45.4%) and the use of cat bibs or bells (31.8%). Local government support was absent for recommendations such as discouraging cruelty to cats and pet insurance and relatively low for informed parasite control (4.6%), providing suitable nutrition (4.6%), enrichment (9.1%), improved disease awareness and prevention (18.2%), improved understanding and provision for cat needs (18.18%), and vaccination (22.7%).

iii. Non-profit organisations (1 Australia, 1 New Zealand, and 1 USA webpage)

Only three webpages were authored by non-profit organisations. All sites supported registration and microchipping, enrichment, vaccination, and desexing, while most supported providing suitable nutrition (66.7%), informed parasite control (66.7%), improved disease awareness and prevention (66.7%), improved understanding and provision for cat needs (66.7%) and discouraging cat abandonment and/or encouraging full adoption (66.7%). Non-profit organisation support was absent for the use of cat bibs and/or bells to discourage hunting and for discouraging cruelty to cats.

iv. Charities (1 Singapore, 3 Australia, 4 New Zealand, 3 UK, and 1 Canada webpage)

Most charities supported improved understanding and provision for cat needs (66.7%), improved disease awareness and prevention (66.7%), desexing (58.3%), and containment (58.3%). They did not support a maximum number of cats per household or identification of cats, and only a minority advocated ethical breeding practices (8.3%) or pet insurance (8.3%).

v. Businesses (3 Australia, 2 UK, and 4 USA webpages)

Most businesses supported providing suitable nutrition (66.7%), improved disease awareness and prevention (66.67%), and enrichment (55.6%).

vi. Personal blogs (1 Australia)

The single personal blog supported providing suitable nutrition, improved understanding and provision for cat needs, improved disease awareness and prevention, and enrichment for cats.

vii. Academic papers (1 Singapore, 2 Australia, 1 UK, and 1 Canada)

The four academic papers revealed in this online search were limited to academic surveys and therefore presented no explicit recommendations towards responsible cat ownership.

viii. Direct veterinary associations (2 Australia, 1 New Zealand, 1 UK, and 2 USA)

Webpages with a direct veterinary association supported registration and microchipping (83.3%), providing suitable nutrition (83.3%), improving understanding and provision for cat needs (83.33%), and enrichment (83.3%). Few recommended ethical breeding practices (16.7%).

Web pages from particular countries also showed clear patterns in recommendations for responsible ownership (Table 6, Figure 3).

Australia (34 webpages)

Most Australian sites advocated registration and microchipping (82.4%), containment (79.4%), and desexing (73.4%).

New Zealand (8 webpages)

Most New Zealand sites supported desexing (87.5%), containment (75%), and registration and microchipping (62.5%). There was limited support for a maximum number of cats per household (12.5%).

UK (7 webpages)

Half (50%) of all webpages discouraging cruelty to cats were from the United Kingdom. Most UK sites advocated for improved understanding and provision for cat needs (71.4%). A relatively low percentage of UK sites recommended identification of cats (14.3%), registration and microchipping (14.3%), pet insurance (14.3%), improved disease awareness and prevention and/or advocacy for veterinary examination (14.3%), and discouraging abandonment and/or encouraging adoption (14.3%).

USA (5 webpages)

Most USA webpages supported enrichment (100%), improved disease awareness and prevention (80%), improved understanding and provision for cat needs (60%), informed parasite control (60%), providing suitable nutrition (60%), and desexing (60%). Fewer sites advocated for containment (20%), discouraging abandonment, and/or encouraging adoption (20%).

Canada (2 webpages)

Both Canadian webpages recommended desexing, registration, and microchipping, as well as improved disease awareness and prevention. One site advocated for providing suitable nutrition and improved understanding and provision for cat needs.

Singapore

Of the two Singaporean webpages, only one was advisory, recommending desexing, improved disease awareness and prevention, improved understanding, and provision for cat needs; including suitable nutrition, ethical breeding practices, and enrichment.

## 4. Discussion

### 4.1. Similar Webpages Occur in Searches, in English, for “Responsible Cat Ownership” across Multiple Countries

Although we considered the top 20 webpages retrieved in Google searches for “responsible cat ownership” from 10 different countries, overall we found only 58 different web pages originating from just six countries. Given that our search approach used a VPN to ensure that each search was made from a specific geographic location and that we cleared all caches and cookies between searches, this finding indicates a small core of English webpages on the topic, irrespective of where in the world one might search. Nevertheless, webpages originating in a particular country were most likely to appear in searches from within that country, allowing for a distinct national tone to recommendations, in line with studies indicating varying national emphases in cat husbandry [3]. The preponderance of webpages from Australia and New Zealand, which have high endemism, may indicate the specific problems caused in these countries by introduced carnivores. It may also mean that advice from Australian and New Zealand pages is insensitive to local nuances in other countries.

### 4.2. Authorship Analysis across Webpages Indicates Who Is Giving Advice

Irrespective of the country where a search originated, local government webpages were the most common author affiliation. This may be linked to the regulatory emphasis noted in Australian and NZ webpages (collectively contributing to 72.5% of search results). This aligns well with increased government regulation of cat ownership in Oceania [33,34].

When only different web pages were considered, patterns of authorship differed across the countries of origin. Most webpages originating in Australia were from local governments. In New Zealand and the UK, most webpages were from charities, while in the US, most were from businesses. Thus, although the large number of local government sites from Australia, with a regulatory focus, were prominent irrespective of where in the world a search originated, there is clear evidence of differing national emphases, although the number of webpages from these countries is comparatively small. This is consistent with studies confirming different emphases on cat husbandry practices in different countries [3].

### 4.3. What Actions Are Recommended and Who Recommends Them?

Searches from all countries found that registration and microchipping, desexing, and containment were recommended by over half the webpages, skewed by the predominance of local government and Australian webpages online. Online local government advice is primarily concerned with administering and applying local laws. For example, Australia has regulations for pet cat management implemented in five of its seven states and territories, with cat containment suburbs implemented in the Australian Capital Territory and cat curfews in areas of South Australia and Victoria [33,34].

In the research literature, if not in the results for online advice from all countries, registration and microchipping are widely accepted as benefiting cat welfare or control (e.g., [35,36,37]). They aid in the return of lost or injured animals and are often seen as less intrusive than collars or tags [35,38]. Despite the lack of consensus about the optimum age to desex cats [39], most veterinary professionals promote surgical desexing to control cat populations because it is permanent and confers behavioural and health benefits [40,41], as well as reducing the social costs of unowned animals [42]. Containment remains highly contentious, and cat owners show variable commitment to it between and within countries [43]. Uncontrolled outdoor access is a major concern for cat welfare by increasing the risk of vehicle collisions, exposure to disease from other animals, and predation [43], while in at least some contexts it is a concern for wildlife predation [44,45]. Conversely, containment restricts some natural behaviours and is linked to health and behavioural issues [46,47]. Consequently, to promote cat welfare (i.e., welfare based around the five freedoms [48]), behavioural restrictions imposed by indoor containment must be counterbalanced by environmental and behavioural enrichment [48]. Despite endorsements of containment, advocation for enrichment was not as prominent online.

While government webpages were strong advocates for registration, microchipping, and desexing, there was little evidence of other recommendations for individual cat welfare. In contrast, non-profit organisations, charities, businesses, and the single personal blog all emphasised improved understanding and provision for cat needs and improved disease awareness and prevention. Additionally, businesses, non-profit organisations, and the personal blog advocated behavioural enrichment for cats. Webpages with direct veterinary associations also supported these recommendations and—like charities, businesses, and personal blogs—did not endorse a maximum number of cats per household.

When webpages were considered by country of origin, national differences appeared. In Australia, there was majority support for microchipping, containment, and desexing. New Zealand was similar, but also had strong support for discouraging cat abandonment or encouraging adoption and lower support for a maximum number of cats per household. This likely reflects minor differences in local laws between the two nations, which may be linked to the increased popularity of cats in NZ [5]. Unlike the Oceanian nations, all other countries endorsed improved understanding and provision for cat needs. Furthermore, non-Oceanian nations also showed little to no support for containment, a maximum number of cats per household, or the identification of cats. This suggests that amongst the English-language advice, there is a clear focus in Australia and NZ on the management of companion cat populations as distinct from an international online concern for individual cats. This may be partially explained by the significantly higher agreement in Australia and NZ that cats pose a substantial threat to valued wildlife in cities, towns, and rural areas [3]. Multiple factors are informing attitudes towards cat management. As testimony to this, a relatively high emphasis on discouraging cruelty to cats was observed on UK webpages. This may correlate with an increased national concern for cat cruelty because UK citizens want to let their cats roam freely [3].

### 4.4. Is the Advice Reliable?

All the recommendations reported on the webpages are supported by the peer-reviewed literature. Publications by veterinary and animal health professionals focus strongly on pet health and welfare (e.g., [49,50], including recommendations for fulfilling cats’ needs if they are contained on the owner’s property, e.g., [43,49,51,52,53,54]. Nevertheless, two recommendations stand out as controversial: desexing and containment.

Veterinary education to improve public awareness of the health benefits of desexing is internationally incentivised [17,50,55]. This is in line with a utilitarian approach to animal ethics—that which prioritises the best aggregate consequences of welfare for all beings involved—and not an animal rights or deontological approach, which argues that animals have intrinsic rights independent of human interests [56]. From a deontological standpoint, routine desexing is ethically problematic because it infringes on an animal’s right to bodily integrity—especially as it cannot be voluntarily relinquished [56]. The consensus for desexing as a responsible ownership practice in many of the surveyed nations may indicate the success of education in promoting its efficacy to control population growth [14] or its value in managing unwanted behaviour [57] and disease [58]. However, it is likely that support for desexing could vary with greater access to more opinions from different regions where deontological consideration may predominate (for example, other areas of Asia or southern Europe).

Advocates for containment prioritise concern for wildlife [59] or the welfare problems of free-ranging cats [60]; some authors argue for both [61,62]. A higher proportion of recommendations for containment in Australia and New Zealand may indicate national concern for the environment or cat health risks such as traffic [1,2,63]. A prioritised concern for wildlife could indicate a utilitarian standpoint, emphasising the welfare of multiple native prey species over domestic cat freedoms. In contrast, a primary concern for cat welfare could indicate a contractarian or relational approach—where the welfare of certain animals is dependent upon human relationships [56]. Equally possible is a shared concern for the welfare of all animals affected—a deontological standpoint or a hybridisation of ethical approaches. Those disagreeing with containment may also be arguing from a perspective that cats should be free to roam in most circumstances [64,65] or from the practical reality of managing indoor cats [52]. From a personal perspective, we find the contention over containment puzzling given the precedent for containment established with many other companion animals and the benefits for cat longevity [23,24], although we acknowledge the need for special consideration of the environmental needs of contained cats [50,62] and problems such as inappropriate elimination in indoor cats [52].

## 5. Limitations

Recommendations for responsible ownership may exclude actions that the author believes are axiomatic and hence unworthy of mention. For example, we could not find recommendations that desexing should only be performed by a veterinarian, although few would disagree. Recommendations regarding such practices as desexing or containment may, for some authors at least, be equally axiomatic and so are not mentioned on webpages on the assumption that everyone is doing them. Thus, North American webpages, for example, may not mention containment given the high profile it has been given by the Humane Society of the US. Authors may also disagree with including mitigation of risks that a cat may pose to the community, other animals, or the environment in a consideration of responsible ownership.

The study used only a single search term, “responsible cat ownership,” in a deliberate attempt to evaluate only sites that addressed the husbandry and welfare of cats as well as wider issues of legislative compliance and social and environmental considerations. Thus, many sites focusing on aspects of cat husbandry alone were excluded, but they might have been detected using a wider range of search terms. Similarly, the study considered only searches in English. There may be information available in other languages that is more likely to be accessed by people searching within some regions. Such an expanded approach might reveal variations arising from cultural differences between countries [3]. Nevertheless, the study demonstrates a method that is expandable to more languages and search terms.

## 6. Conclusions

Online advice in English surrounding responsible cat ownership is dominated by local government, mainly from Australia, advocating registration and microchipping, desexing, and containment rather than environmental and behavioural enrichment in households. In contrast to Oceania, other nations supported recommendations around individual cat welfare. These findings support those of Hall et al. [3], showing how divergent welfare considerations inform attitudes to cat ownership internationally and, perhaps, what people consider should be part of a definition of responsible ownership. Local considerations may drive a gap between responsible ownership as we have defined it and what is practised in different communities.

While local communities should choose the management approaches they wish, it seems that the major motivator driving husbandry in many countries is cat welfare. Despite its substantial benefits for increasing the longevity of cats and reducing problems of trauma and disease, containment is not high on the list of recommendations for responsible ownership in several countries. This may result from cultural differences across the countries surveyed, which could be teased out in follow-up work exploring a wider range of search terms and languages. Possibilities include, but are not limited to, searches for behavioural problems related to cat ownership, recommendations for feeding and veterinary care, specific advice about desexing, and regulations/legislation. Exploring online information rather than using interviews or questionnaires is an effective way of determining what information is available and, if searches extend into exploring social media posts, can also indicate individuals’ opinions and practices.

## Figures and Tables

**Table 1:** Overview of the search strategy.

Date of Search	Country (City of Search and Population)	Number of New Webpages in First 20 Search Results/Numbers of Duplicate Results and Country Where Duplicate Webpage Initially Identified	Running Total of Different Pages	Number of Unique Pages
1 January 2022	Australia (Perth, 1.9 million)	20	20	7
1 January 2022	USA (Seattle, 4.1 million)	12/8(AUS)	32	4
1 January 2022	Singapore (Singapore, 5.6 million)	4/12(AUS), 4(USA)	36	0
1 January 2022	UK (London, 9.5 million)	8/7(AUS), 5(USA)	44	6
4 January 2022	South Africa (Johannesburg, 6.1 million)	6/6(AUS), 6(USA), 1(SGP), 1(GBR)	50	1
4 January 2022	NZ (Wellington, 0.2 million)	6/5(AUS), 3 (USA), 3(ZAF) 2(SGP), 1(GBR)	56	6
4 January 2022	Brazil (Brasilia, 4.8 million)	0/9(AUS), 5(USA), 3(ZAF), 2(SGP), 1(GBR)	56	0
4 January 2022	Israel (Tel Aviv, 4.4 million)	2/7(AUS), 5(USA), 4(ZAF), 2(SGP)	58	0
4 January 2022	Italy (Rome, 4.3 million)	0/9(AUS), 5(USA), 4(ZAF), 1(SGP), 1(ISR)	58	0
4 January 2022	Egypt (Cairo, 21.8 million)	0/7(AUS), 5(USA), 4(ZAF), 2(SGP), 2(ISR)	58	0

**Table 2:** For each geographic region, sub-region, and country of search, the data indicate the number and percentage of search results by country of webpage origin.

Region	Sub-Region	Country of Search	Number and % of Results by Country of Webpage Origin
Oceania	Australia and NZ	Australia NZ	AUS: 18 (90%), USA: 2 (10%) AUS: 10 (50%), NZL: 7 (35%), USA: 1 (5%), GBR: 1 (5%), CAN: 1 (5%)
Americas	North America	USA	AUS: 10 (50%), USA: 5 (25%), SGP: 2 (10%), NZL: 1 (5%), GBR: 1 (5%), CAN: 1 (5%)
	South America	Brazil	AUS: 12 (60%), NZL: 3 (15%), USA: 2 (10%), GBR: 1 (5%), CAN: 1 (5%), SGP: 1 (5%)
Europe	Northern Europe	UK	AUS: 10 (50%), GBR: 7 (35%), USA: 2 (10%), SGP: 1 (5%)
	Southern Europe	Italy	AUS: 13 (65%), NZL: 2 (10%), USA: 2 (10%), GBR: 1 (5%), CAN: 1 (5%), SGP: 1 (5%)
Asia	Western Asia	Israel	AUS: 12 (60%), NZL: 3 (15%), USA: 2 (10%), GBR: 1 (5%), CAN: 1 (5%), SGP: 1 (5%)
	South-eastern Asia	Singapore	AUS: 14 (70%), USA: 2 (10%), GBR: 1 (5%) CAN: 1 (5%), NZL: 1 (5%), SGP: 1 (5%)
Africa	Southern Africa	South Africa	AUS: 10 (50%), NZL: 3 (15%) USA: 2 (10%), GBR: 2 (10%), SGP: 2 (10%), CAN: 1 (5%)
	Northern Africa	Egypt	AUS: 12 (60%), NZL: 3 (15%), USA: 2 (10%), SGP: 1 (5%), GBR: 1 (5%), CAN: 1 (5%)
All	All	All	AUS:121 (60.5%), NZL: 24 (12%), USA: 22 (11%), GBR: 16 (8%), SGP: 10 (5%), CAN: 7 (3.5%)

**Table 3:** No. of webpages recommending 16 identified “responsible cat ownership” practices by country of search.

Recommendation	Country of Search										
	Australia	NZ	USA	Brazil	UK	Italy	Singapore	Israel	S. Africa	Egypt	All
Environmental and behavioural enrichment	6	7	10	8	6	7	6	9	9	9	77
Containment	17	15	10	12	9	12	13	12	11	12	123
Discouraging abandonment, encouraging full adoption of strays/rescue cats	4	6	3	3	4	3	3	2	2	3	33
Ethical breeding	4	3	3	4	2	5	3	4	3	5	36
Providing for cat behavioural and physical needs	5	8	11	10	7	10	7	12	11	12	93
Improved disease control/advocacy for veterinary checks	9	9	13	12	7	10	10	12	10	12	104
Maximum number of cats/household	6	2	2	5	3	6	4	5	2	5	40
Parasite control	7	4	7	6	6	4	7	6	4	6	57
Pet insurance	0	1	1	3	1	1	2	3	2	3	17
Suitable nutrition	5	6	10	8	8	7	6	9	8	6	76
Registration and microchipping	18	15	13	17	10	16	18	16	14	16	153
Desexing	16	16	12	14	7	14	15	14	12	14	134
Discourage hunting (cat bibs or bells)	2	6	1	2	2	2	2	2	2	2	23
Vaccination	9	7	8	7	6	5	7	7	5	7	68
Identification on cat	8	6	4	7	5	8	6	7	6	7	64
Discouraging cruelty to cats	0	3	0	1	2	1	0	1	1	1	10

**Table 4:** The country of origin and authorship of 58 different webpages identified in the searches. These webpages could have been found in a search from any country.

Authorship	Country of Webpage Origin					
	Singapore	Australia	New Zealand	UK	USA	Canada
Federal or state government	0	5	1	1	0	0
Local government	0	19	2	0	0	0
Non-profit organisation	0	1	1	0	1	0
Charity	1	3	4	3	0	1
Business	0	3	0	2	4	0
Personal blog	0	1	0	0	0	0
Academic paper	1	2	0	1	0	1
Veterinary	0	2	1	1	2	0
No. of webpages *	2	34	8	7	5	2

* Note that authors may belong to more than one authorship category, so the total number of authors for a country may exceed the number of webpages from that country.

**Table 5:** The recommendations across the 58 different webpages identified in the searches. A single webpage may, of course, make multiple recommendations.

Recommendation	No. of Different Webpages	Percentage of Different Webpages
Environmental and behavioural enrichment	17	29.3
Containment	35	60.3
Discouraging abandonment, encouraging full adoption of strays/rescue cats	11	19.0
Ethical breeding	10	17.2
Providing for cat behavioural and physical needs	23	40.0
Improved disease control/advocacy for veterinary checks	22	38.0
Maximum number of cats/household	13	34.2
Parasite control	11	19.0
Pet insurance	3	5.2
Suitable nutrition	20	34.5
Registration and microchipping	38	65.5
Desexing	38	65.5
Discourage hunting (cat bibs or bells)	10	17.2
Vaccination	17	29.31
Identification on cat	19	32.8
Discouraging cruelty to cats	6	10.4

**Table 6:** The recommendations across the country of origin of the 58 different webpages identified in the searches.

Recommendation	Country of Webpage Origin					
	Singapore	Australia	New Zealand	UK	USA	Canada
Environmental and behavioural enrichment	1	8	3	0	5	0
Containment	0	27	6	0	1	2
Discouraging abandonment, encouraging full adoption of strays/rescue cats	0	5	6	0	1	2
Ethical breeding	1	6	2	1	0	0
Providing for cat behavioural and physical needs	1	9	4	5	3	1
Improved disease control/advocacy for veterinary checks	1	10	4	1	4	2
Maximum no. of cats/household	0	12	1	0	0	0
Parasite control	0	6	2	0	3	0
Pet insurance	0	0	2	1	0	0
Suitable nutrition	1	9	3	3	3	1
Registration and microchipping	0	28	5	1	2	2
Desexing	1	25	7	0	3	2
Discourage hunting (cat bibs, bells)	0	6	4	0	0	0
Vaccination	0	12	3	0	2	0
Identification on cat	0	16	2	1	0	0
Discouraging cruelty to cats	0	1	2	3	0	0
No. of webpages *	2	34	8	7	5	2

* A webpage may make multiple recommendations, so the total number of recommendations for a country exceeds the number of webpages from that country.

## Data Availability

Relevant data are included in the relevant tables and figures in the paper and the Supplementary Materials.

## Supplementary Materials

The following supporting information can be downloaded at: https://www.mdpi.com/article/10.3390/ani13152434/s1, S1: Access details for the 58 different webpages.

## References

[1] Crawford H.M., Calver M.C., Fleming P.A. (2019). A Case of Letting the Cat out of The Bag—Why Trap-Neuter-Return Is Not an Ethical Solution for Stray Cat (*Felis catus*) Management. Animals.

[2] Carter J., Paterson M.B.A., Morton J.M., Gelves-Gomez F. (2020). Beliefs and Attitudes of Residents in Queensland, Australia, about Managing Dog and Cat Impacts on Native Wildlife. Animals.

[3] Hall C.M., Adams N.A., Bradley J.S., Bryant K.A., Davis A.A., Dickman C.R., Fujita T., Kobayashi S., Lepczyk C.A., McBride E.A. (2016). Community Attitudes and Practices of Urban Residents Regarding Predation by Pet Cats on Wildlife: An International Comparison. PLoS ONE.

[4] Dawson L., Cheal J., Niel L., Mason G. (2019). Humans can identify cats’ affective states from subtle facial expressions. Anim. Welf..

[5] Zealand C.A.N. (2020). Companion Animals in New Zealand 2016.

[6] Seo A., Ueda Y., Tanida H. (2021). Health Status of ‘Community Cats’ Living in the Tourist Area of the Old Town in Onomichi City, Japan. J. Appl. Anim. Welf. Sci..

[7] Statista Most Popular Pets in Households in Russia in 2021. https://www.statista.com/statistics/1256042/most-popular-pets-russia/.

[8] Sims V., Evans K.L., Newson S.E., Tratalos J.A., Gaston K.J. (2008). Avian assemblage structure and domestic cat densities in urban environments. Divers. Distrib..

[9] Lepczyk C.A., Calver M.C. (2022). Cat got your tongue? The misnomer of ‘community cats’ and its relevance to conservation. Biol. Invasions.

[10] Kohl P.A., Collins S.J., Eichholz M. (2020). Metaphor, Trust and Support for Non-native Species Control. Environ. Commun..

[11] Wald D.M., Jacobson S.K., Levy J.K. (2013). Outdoor cats: Identifying differences between stakeholder beliefs, perceived impacts, risk and management. Biol. Conserv..

[12] Farnworth M.J., Campbell J., Adams N.J. (2011). What’s in a Name? Perceptions of Stray and Feral Cat Welfare and Control in Aotearoa, New Zealand. J. Appl. Anim. Welf. Sci..

[13] Rijks J., Cito F., Cunningham A., Rantsios A., Giovannini A. (2016). Disease Risk Assessments Involving Companion Animals: An Overview for 15 Selected Pathogens Taking a European Perspective. J. Comp. Pathol..

[14] Costa E.D., Martins C.M., Cunha G.R., Catapan D.C., Ferreira F., Oliveira S.T., Garcia R.D.C.M., Biondo A.W. (2017). Impact of a 3-year pet management program on pet population and owner’s perception. Prev. Veter-Med..

[15] Day M. (2016). The CALLISTO Project: A Summary. J. Comp. Pathol..

[16] Elliott A., Howell T.J., McLeod E.M., Bennett P.C. (2019). Perceptions of Responsible Cat Ownership Behaviors among a Convenience Sample of Australians. Animals.

[17] Gunaseelan S., Coleman G.J., Toukhsati S.R. (2013). Attitudes toward Responsible Pet Ownership Behaviors in Singaporean Cat Owners. Anthrozoos.

[18] Ryan M.J., Worthngton A.K., Worthington A.K. (2021). Theory of planned behaviour. Persuasion Theory in Action: An Open Educational Resources.

[19] Dabritz H.A., Miller M.A., Gardner I.A., Packham A.E., Atwill E.R., Conrad P.A. (2008). Risk Factors for Toxoplasma gondii Infection in Wild Rodents from Central Coastal California and a Review of T. gondii Prevalence in Rodents. J. Parasitol..

[20] Toukhsati S., Bennett P.C., Coleman G.J. (2007). Behaviors and Attitudes towards Semi-Owned Cats. Anthrozoos.

[21] Hood J. Pet ownership and Asian multiculturalism. Proceedings of the Seventh National Conference on Urban Animal Management in Australia.

[22] Linklater W.L., Farnworth M.J., Heezik Y., Stafford K.J., Macdonald E.A. (2019). Prioritizing cat-owner behaviors for a campaign to reduce wildlife depredation. Conserv. Sci. Pract..

[23] Moreau D., Cathelain P., Lacheretz A. (2003). Comparative study of causes of death and life expectancy in carnivorous pets (II). Rev. Med. Vet..

[24] Loyd K.A.T., Hernandez S.M., Abernathy K.J., Shock B.C., Marshall G.J. (2013). Risk behaviours exhibited by free-roaming cats in a suburban US town. Veter-Rec..

[25] Grieshofer T. (2022). Lay Advisers in Family law Settings: The Role and Quality of Advice Provided on Social media. Soc. Leg. Stud..

[26] Miller T., Stockley R., Drummond A., Watkins C., Georgiou R., Ahuja K.D.K., Bird M.-L. (2022). Online advice for the symptomatic management of post-stroke fatigue: A scoping review. J. Psychosom. Res..

[27] Belot M., Kircher P., Muller P. (2019). Providing Advice to Jobseekers at Low Cost: An Experimental Study on Online Advice. Rev. Econ. Stud..

[28] Jokar M., Rahmanian V., Sharifi N., Rahmanian N., Khoubfekr H. (2021). Feline Infectious Peritonitis and Feline Coronavirus Interest During the COVID-19 Pandemic: A Google Trends Analysis. Am. J. Anim. Veter-Sci..

[29] Pung W.C., Ho A.P. (2022). A Comparative Study on Online Advice-Seeking Strategies between Malaysian and Australian Women. GEMA Online J. Lang. Stud..

[30] Fokkema T., de Vos R.-J., van Ochten J.M., Verhaar J.A., Davis I.S., Bindels P.J., Bierma-Zeinstra S.M., van Middelkoop M. (2017). Preventing running-related injuries using evidence-based online advice: The design of a randomised-controlled trial. BMJ Open Sport Exerc. Med..

[31] Gunaratne J., Zalmanson L., Nov O. (2018). The Persuasive Power of Algorithmic and Crowdsourced Advice. J. Manag. Inf. Syst..

[32] Sarah V. The Value of Google Result Positioning. https://research.chitika.com/the-value-of-google-result-positioning-2/.

[33] Legge S., Nou T. (2021). The Management of Cats by Local Governments in Australia: Summary of National Survey Results. Project 7.4 of the National Science Programme’s Threatened Species Recovery Hub; Brisbane. https://www.nespthreatenedspecies.edu.au/media/2rxhzg1o/7-4-the-management-of-cats-by-local-governments-in-australia-summary-of-national-survey-results-ff_v2.pdf.

[34] ACT Government (2021). ACT Cat Plan 2021–2031. A Plan Developed under the 2017 ACT Animal Welfare and Management Strategy.

[35] Lord L.K., Griffin B., Slater M.R., Levy J.K. (2010). Evaluation of collars and microchips for visual and permanent identification of pet cats. J. Am. Vet. Med. Assoc..

[36] Lord L.K., Wittum T.E., Ferketich A.K., Funk J.A., Rajala-Schultz P.J. (2007). Search methods that people use to find owners of lost pets. J. Am. Vet. Med. Assoc..

[37] Lord L.K., Wittum T.E., Ferketich A.K., Funk J.A., Rajala-Schultz P.J. (2007). Search and identification methods that owners use to find a lost cat. J. Am. Veter-Med. Assoc..

[38] Calver M., Adams G., Clark W., Pollock K. (2013). Assessing the safety of collars used to attach predation deterrent devices and ID tags to pet cats. Anim. Welf..

[39] Farnworth M.J., Adams N.J., Seksel K., Waran N.K., Beausoleil N.J., Stafford K.J. (2013). Veterinary attitudes towards pre-pubertal gonadectomy of cats: A comparison of samples from New Zealand, Australia and the United Kingdom. N. Z. Veter-J..

[40] Murray J.K., Mosteller J.R., Loberg J.M., Andersson M., Benka V.A.W. (2015). Methods of fertility control in cats: Owner, breeder and veterinarian behavior and attitudes. J. Feline Med. Surg..

[41] Spain C.V., Scarlett J.M., Houpt K.A. (2004). Long-term risks and benefits of early-age gonadectomy in cats. J. Am. Veter-Med. Assoc..

[42] Stavisky J., Brennan M.L., Downes M., Dean R. (2012). Demographics and economic burden of un-owned cats and dogs in the UK: Results of a 2010 census. BMC Veter-Res..

[43] Tan S.M., Stellato A.C., Niel L. (2020). Uncontrolled Outdoor Access for Cats: An Assessment of Risks and Benefits. Animals.

[44] Loss S.R., Will T., Marra P.P. (2013). The impact of free-ranging domestic cats on wildlife of the United States. Nat. Commun..

[45] Walker J.K., Bruce S.J., Dale A.R. (2017). A Survey of Public Opinion on Cat (*Felis catus*) Predation and the Future Direction of Cat Management in New Zealand. Animals.

[46] Amat M., de la Torre J.L.R., Fatjó J., Mariotti V.M., Van Wijk S., Manteca X. (2009). Potential risk factors associated with feline behaviour problems. Appl. Anim. Behav. Sci..

[47] Rowe E., Browne W., Casey R., Gruffydd-Jones T., Murray J. (2015). Risk factors identified for owner-reported feline obesity at around one year of age: Dry diet and indoor lifestyle. Prev. Veter-Med..

[48] Rochlitz I. (2003). Study of factors that may predispose domestic cats to road traffic accidents: Part 1. Veter-Rec..

[49] Rochlitz I. (2005). A review of the housing requirements of domestic cats (*Felis silvestris* catus) kept in the home. Appl. Anim. Behav. Sci..

[50] De Andrade F.T.M., De Araújo C.L., Paulo O.L.D.O.H., Rocha J.R., Dias F.G.G., Pereira L.D.F., Jorge A.T., Honsho C.S. (2015). Responsible pet ownership: A multidisciplinary issue. Acta Vet. Bras..

[51] Rochlitz I. (2003). Study of factors that may predispose domestic cats to road traffic accidents: Part 2. Veter-Rec..

[52] Rochlitz I., Turner D.C., Bateson P. (2014). Feline welfare issues. The Domestic Cat: The Biology of its Behaviour.

[53] Ellis S.L.H., Rodan I., Carney H.C., Heath S., Rochlitz I., Shearburn L.D., Sundahl E., Westropp J.L. (2013). AAFP and ISFM Feline Environmental Needs Guidelines. J. Feline Med. Surg..

[54] Machado D.d.S., Gonçalves L.d.S., Vicentini R.R., Ceballos M.C., Sant’anna A.C. (2020). Beloved Whiskers: Management Type, Care Practices and Connections to Welfare in Domestic Cats. Animals.

[55] Finkler H., Terkel J. (2012). The contribution of cat owners’ attitudes and behaviours to the free-roaming cat overpopulation in Tel Aviv, Israel. Prev. Veter-Med..

[56] Sandøe P., Corr S., Palmer C. (2015). Companion Animal Ethics.

[57] Voith V.L. (2009). The Impact of Companion Animal Problems on Society and the Role of Veterinarians. Veter-Clin. N. Am. Small Anim. Pract..

[58] Machado D., Machado J.C., De Souza J.O.T., Sant’Anna A.C. (2019). The importance of responsible cat ownership: Practical aspects and connections with animal welfare. Rev. Acad. Cienc. Anim..

[59] Mori E., Menchetti M., Camporesi A., Cavigioli L., De Fatis K.T., Girardello M. (2019). License to Kill? Domestic Cats Affect a Wide Range of Native Fauna in a Highly Biodiverse Mediterranean Country. Front. Ecol. Evol..

[60] Calver M.C., Crawford H.M., Fleming P.A. (2020). Response to Wolf et al.: Furthering Debate over the Suitability of Trap-Neuter-Return for Stray Cat Management. Animals.

[61] McLeod L.J., Driver A.B., Bengsen A.J., Hine D.W. (2017). Refining Online Communication Strategies for Domestic Cat Management. Anthrozoos.

[62] McLeod L.J., Hine D.W., Bengsen A.J., Driver A.B. (2017). Assessing the impact of different persuasive messages on the intentions and behaviour of cat owners: A randomised control trial. Prev. Veter-Med..

[63] Clarke A.L., Pacin T. (2002). Domestic cat “colonies” in natural areas: A growing exotic species threat. Nat. Areas J..

[64] Abbate C. (2021). Re-defending Feline Liberty: A Response to Fischer. Acta Anal..

[65] Abbate C. (2020). A Defense of Free-Roaming Cats from a Hedonist Account of Feline Well-being. Acta Anal..

