# Supplementary material for: Piloting an International Comparison of Readily Accessible Online English Language Advice Surrounding Responsible Cat Ownership
Source: Animals (Basel). 2023 Jul 27;13(15):2434. doi: 10.3390/ani13152434 (PMC10416957; doi:10.3390/ani13152434)
Supplement: Supplementary file 1 [file animals-13-02434-s001.zip › animals-2458078-supplementary.pdf]

Supplementary Material S1

URL list for the 58 different webpages. Where a webpage only occurred in a search from one country, that country is shown in a superscript after the relevant url.

1. https://kb.rspca.org.au/knowledge-base/how-can-i-be-a-responsible-cat-owner/
2. https://www.dlgsc.wa.gov.au/local-government/community/cats-and-dogs/laws-for-responsible-cat-owners
3. https://www.claremont.wa.gov.au/MediaLibrary/TownOfClaremont/Documents/WESROC-Cat-Ownership-Brochure.pdf
4. https://www.joondalup.wa.gov.au/wp-content/uploads/2017/12/Responsible-Cat-Ownership.pdf
5. https://www.rspcansw.org.au/blog/animal-care-information/cat-owner-top-tips/
6. https://www.bassendean.wa.gov.au/Profiles/bassendean/Assets/ClientData/Document-Centre/Rangers/Responsible_Cat_Ownership_Information_Sheet.pdf
7. https://www.wandering.wa.gov.au/my-property-business/animal-information/responsible-cat-owners.aspx [(AUS)]
8. https://www.cockburn.wa.gov.au/Responsible-Cat-Ownership-And-Nuisance-Cats
9. https://www.ava.com.au/policy-advocacy/policies/companion-animals-management-and-welfare/the-responsible-ownership-of-dogs-and-cats-and-the-human-animal-bond/
10. https://vcahospitals.com/know-your-pet/responsible-cat-ownership
11. https://www.sunshinecoast.qld.gov.au/Living-and-Community/Animals-and-Pets/Responsible-Cat-Ownership
12. https://agriculture.vic.gov.au/livestock-and-animals/animal-welfare-victoria/cats/legal-requirements-for-cat-owners
13. https://www.nedlands.wa.gov.au/responsible-cat-ownership-western-suburbs [(AUS)]
14. https://www.wanneroo.wa.gov.au/info/20006/animals_and_pets/70/owning_a_cat [(AUS)]
15. https://animoz.world/stories/how-to-be-a-responsible-cat-owner-in-australia/ [(AUS)]
16. https://www.environment.gov.au/biodiversity/threatened/publications/protecting-our-wildlife-responsible-pet-ownership [(AUS)]
17. https://www.belmont.wa.gov.au/live/at-your-place/pets-and-animals/cat-ownership-rules-and-management [(AUS)]
18. https://www.thesprucepets.com/cats-4162124
19. https://www.moretonbay.qld.gov.au/Services/Animals/Cats
20. https://frontlinepetcare.com.au/responsible-cat-ownership [(AUS)]
21. https://pawesomecats.com/responsible-cat-ownership/
22. https://www.catwelfare.org/responsible-cat-ownership/
23. https://www.avma.org/resources-tools/pet-owners/responsible-pet-ownership [(USA)]
24. https://figopetinsurance.com/blog/cat-ownership-101-guide-new-cat-parents [(USA)]
25. https://www.rover.com/blog/10-tips-help-become-best-cat-owner/ [(USA)]
26. https://ottawahumane.ca/your-pet/animal-tips/cat-owners-responsibilities/ [(USA)]
27. https://www.medivet.co.uk/pet-care/pet-advice/responsible-cat-owner/
28. https://www.ncbi.nlm.nih.gov/pmc/articles/PMC6769723/
29. https://www.cbcity.nsw.gov.au/resident/animals-pets/owning-a-pet/responsible-cat-ownership
30. https://catprotection.org.au/responsible-cat-ownership/
31. https://www.aucklandcouncil.govt.nz/dogs-animals/keeping-other-animals/keeping-cats/Pages/be-responsible-cat-owner.aspx
32. https://www.researchgate.net/publication/255522729_Attitudes_toward_Responsible_Pet_Ownership_Behaviors_in_Singaporean_Cat_Owners
33. https://www.cityservices.act.gov.au/pets-and-wildlife/domestic-animals/cats/your-responsibilities
34. https://www.spca.nz/advice-and-welfare/article/responsible-cat-ownership
35. https://www.kingborough.tas.gov.au/services/animal-management-2/cats-2/responsible-cat-ownership/

36. https://www.halifax.ca/sites/default/files/documents/home-property/animal-services/RespCatOwnership_RackCard2017_fileforWeb.pdf
37. https://www.cats.org.uk/help-and-advice/getting-a-cat/cats-and-the-law (GBR)
38. https://www.nidirect.gov.uk/articles/welfare-cats-introduction (GBR)
39. http://www.thecatgroup.org.uk/pdfs/Cats-law-web.pdf (GBR)
40. https://www.scottishwildcataction.org/supercat/take-the-pledge/ (GBR)
41. https://wildlifescience.org/portfolio/domestic-cats/ (GBR)
42. https://www.innerwest.nsw.gov.au/live/information-for-residents/dogs-and-cats/cats/being-a-responsible-cat-owner (GBR)
43. https://www.brisbane.qld.gov.au/community-and-safety/pets-and-livestock/keeping-a-pet-in-brisbane/keeping-a-cat
44. https://esajournals.onlinelibrary.wiley.com/doi/full/10.1002/fee.2254
45. https://agriculture.vic.gov.au/livestock-and-animals/animal-welfare-victoria/community-and-education/responsible-cat-ownership-course
46. https://www.mdpi.com/2076-2615/9/9/703
47. https://veteducation.com.au/responsible-cat-ownership-to-reduce-cat-impacts-on-wildlife/
48. https://www.nzva.org.nz/resource/companion-animal/cat-ownership/
49. https://wellington.govt.nz/-/media/your-council/plans-policies-and-bylaws/plans-and-policies/a-to-z/domestic-animals/animal-cat-factsheet.pdf
50. https://www.cityservices.act.gov.au/__data/assets/pdf_file/0019/1435006/Cat-ownership-factsheet-2015.pdf
51. https://www.newzealandnow.govt.nz/resources/owning-a-cat (NZ)
52. https://communitylaw.org.nz/community-law-manual/chapter-27-neighbourhood-life/dogs-and-other-animals/cats-care-and-control-of-your-cat/ (NZ)
53. https://www.forestandbird.org.nz/resources/responsible-pet-ownership-guide (NZ)
54. https://www.forestandbird.org.nz/sites/default/files/2018-05/CatFactsheet_forestandbird.pdf (NZ)
55. https://www.brokenhill.nsw.gov.au/Services/Pets-and-animals/Responsible-Cat-Ownership (NZL)
56. https://www.narrogin.wa.gov.au/live/services/ranger-services/responsible-cat-ownership.aspx (NZ)
57. https://www.maranoa.qld.gov.au/downloads/file/1036/responsible-cat-ownership
58. https://karratha.wa.gov.au/sites/default/files/uploads/CATS_Responsible-Cat-Ownership-web.pdf
